# Investigation of combined treatment of acupuncture and neurofeedback for improving cognitive function in mild neurocognitive disorder: A randomized, assessor-blind, pilot study

**DOI:** 10.1097/MD.0000000000027218

**Published:** 2021-09-17

**Authors:** Jin-Hyung Jeong, Changjin Jung, Jieun Kim, Ju-Yeon Kim, Hae Sook Kim, Yang-Chun Park, Jun-Hwan Lee, In Chul Jung

**Affiliations:** a Department of Oriental Neuropsychiatry, College of Korean Medicine, Daejeon University, Daejeon, Republic of Korea; b Clinical Medicine Division, Korea Institute of Oriental Medicine, Republic of Korea; c Clinical trial center, Daejeon Korean Medicine Hospital of Daejeon University, Republic of Korea; d Department of Internal Medicine, Daejeon Korean Medicine Hospital of Daejeon University, Republic of Korea; e Korean Medicine Life Science, University of Science & Technology (UST), Republic of Korea.

**Keywords:** acupuncture, dementia, mild cognitive impairment, mild neurocognitive disorder, neurofeedback

## Abstract

**Background:**

Mild neurocognitive disorder (MND) is an intermediate state that can progress to dementia, and the cognitive reserve of MND is an important task in preventing dementia. Acupuncture and neurofeedback (NF) training have been used to improve cognitive function and treat MND or dementia, but their effectiveness remains controversial. In this trial, we will evaluate the efficacy and safety of combined NF-acupuncture treatment in comparison with single acupuncture treatment.

**Methods and design:**

This study is a randomized, assessor-blind, pilot trial. It is designed in accordance with the Standards for Reporting Interventions in Controlled Trials of Acupuncture. A total of 44 MND participants who meet the inclusion and exclusion criteria will be enrolled, and each will be randomly assigned to 1 of 2 groups of 22 subjects. Each subject will visit 24 times over 12 weeks and receive either acupuncture or NF-acupuncture combined treatment. At visit 25 (week 13), a follow-up evaluation will be performed, and then the investigator will analyze the results. The primary outcome is defined by the Korean version of the Montreal Cognitive Assessment score from screening to visit 25. The secondary outcome includes the following: change in Alzheimer Disease Assessment Scale–Cognitive, the Korean version of the Beck Depression Inventory, Body Awareness Questionnaire, delayed matching to sample task scores, and functional near-infrared spectroscopy values, from visit 1 to visit 25; heart rate variability values from visit 1 to visit 5, visit 9, visit 13, visit 21, visit 25; breath per minute values from visit 1 to visit 1 to 25.

**Discussion:**

We will evaluate the effectiveness and safety of combined NF-acupuncture therapy, and expect that it will serve as the basis for the use of NF together with acupuncture in the clinical setting.

**Trial registration number:**

KCT0004972 (registered in Clinical Research Information Service of the Republic of Korea, https://cris.nih.go.kr/cris/search/detailSearch.do/16239)

## 1 Introduction

Dementia is a neurodegenerative disorder defined as chronic acquired loss of 2 or more cognitive abilities that represent a decline from the prior level of function, resulting in impaired functional abilities in day-to-day life.^[1]^ According to the World Health Organization, worldwide dementia costs were estimated at US$ 818 billion in 2015, and by 2030, it is estimated that the cost of caring for people with dementia will rise to US$ 2 trillion.^[2]^

Mild neurocognitive disorder (MND) is considered an intermediate state between healthy aging and early dementia, or as a potential state that represents a higher risk of dementia.^[3]^ In South Korea, the prevalence rate of MND is 23.7% in people older than 65 years.^[4]^ To date, there have been no drug interventions that delay conversion to dementia. To prevent progression to dementia in MND, various interventions are needed that include decreasing neuropathological damage and maximizing function.^[3]^

To increase and maintain the cognitive reserve, several cognitive training programs have been increasingly used in MND.^[5]^ An increasing number of studies that present complementary and alternative approaches of acupuncture intervention for cognitive reserves have also been reported.^[6,7]^ The afferent neural circuit of somatosensory induced by acupuncture specifically increase cognitive-related functions.^[8]^ Altered brain function and functional networks of cognitive-related brain regions as well as sensorimotor areas following acupuncture in patients with cognitive impairment support the central nervous system mechanism of acupuncture.^[9–11]^

Neurofeedback (NF) training is a noninvasive brain stimulation technique with operant conditioning closed-loop feedback control mechanism, used in the treatment of a range of neurological and psychiatric conditions.^[12]^ Several studies have reported the effectiveness of NF training in improving cognitive function in dementia^[13,14]^ and MND^[15–17]^ patients.

Based on these neural-neurofunctional groundworks, we believe that combined NF-acupuncture treatment could be a meaningful approach to effectively improve cognitive function. However, due to the lack of clinical research, we plan to conduct a randomized, assessor-blind pilot study.

## 2 Methods

### 2.1 Objectives

The main objective of this pilot study is to evaluate the efficacy and safety of NF-acupuncture combined therapy compared to acupuncture single treatment in MND patients.

### 2.2 Study design and setting

This study is designed as a randomized, assessor-blind, parallel trial. The clinical trial site will be set as Daejeon Korean Medicine Hospital of Daejeon University in the Republic of Korea. The number of eligible participants will be set as 44. Subjects will be divided into 2 equal groups: the combined treatment group (experimental group) and the single treatment group (control group). Each group will be treated twice a week for 12 weeks (visits 1–24). After visit 24, during the follow-up period, participants will be guided to visit for follow-up assessment, which will be visit 25. The primary outcome is the Korean version of Montreal Cognitive Assessment (MoCA-K)^[18]^ score. The secondary outcomes include Alzheimer Disease Assessment Scale–Cognitive (ADAS-Cog)^[19]^ subscale scores, the Korean version of Beck Depression Inventory (K-BDI)^[20]^ score, Body Awareness Questionnaire (BAQ)^[21]^ score, breath per minute (BPM), heart rate variability (HRV) values measured during NF training,^[22]^ functional near-infrared spectroscopy (fNIRS)^[23]^ value, and delayed matching to sample task (DMTS)^[24]^ score. This protocol (version 1.3, July 31, 2020) follows the Recommendations for Standard Protocol Items: Recommendations for Interventional Trial guidelines. The final approval date of clinical research information service registration is April 28, 2020.

### 2.3 Participants

#### 2.3.1 Recruitment

Recruitment will be started on April 22, 2020 (the anticipated date of first enrollment is May 15, 2020, and completed by November 30, 2021. The recruitment period may be extended depending on registration. A consent document that contains information about the background, purpose, intervention, outcome, benefit, and adverse events will be given to all participants or their surrogates. The investigating doctor will explain the consent fully before registration and notify all subjects about the option to withdraw without any penalty.

#### 2.3.2 Screening

Subjects who have received sufficient explanation and voluntarily signed the consent form will be given a screening number (ie, DJNP-20-01-S010). The investigator will elevate eligibility by applying the inclusion and exclusion criteria, conducting vital sign checks and laboratory tests, taking demographic information and medical history of the subject. The investigator will open the envelope that contains the randomization code and give the subject an identification code containing the stratification number (ie, DJNP-20-01-R1210).

##### 2.3.2.1 Inclusion criteria

1. Males and females aged between 50 and 75 years.
2. Participants diagnosed with MND based on the diagnostic and statistical manual of mental disorders-5 diagnostic criteria.
3. Participants with MoCA-K score of 22 or less.
4. Participants who have a level of education of more than 6 years (more than elementary education level).
5. Participants or authorized surrogates who voluntarily sign the clinical trial consent form.

##### 2.3.2.2 Exclusion criteria

1. Alzheimer disease, vascular dementia, Parkinson disease, Huntington disease, hydrocephalus, etc.
2. Patients who have had major psychiatric disorders such as schizophrenia, delusional disorder, bipolar disorder, alcohol, or substance abuse disorder, were diagnosed according to diagnostic and statistical manual of mental disorders-5 diagnostic criteria.
3. Patients who have had other neurologic diseases such as epilepsy, brain injury, stroke, etc.
4. Patients who have participated in other interventional clinical trials for 1 month before the start of the trial.
5. Patients who have performed the diagnostic test of cognitive impairment 3 months before the start of the trial.
6. Patients who are pregnant, lactating, or not using appropriate methods of contraception.
7. Patients with unstable medical conditions (investigators will judge these conditions according to the standard work procedure, based on the results of vital signs, clinical pathology, electroencephalography (EEG), and chest radiography, etc).
8. Patients with hypertension, as defined by a systolic blood pressure above 160 mm Hg, or diastolic blood pressure above 95 mm Hg.
9. Patients who had clinically significant liver disease or had serum aspartate aminotransferase, alanine aminotransferase, and/or gamma-glutamyl transpeptidase values more than twice the laboratory test upper limit reference.
10. Patients who cannot be measured by fNIRS.
11. Patients who disagree with acupuncture treatment.
12. Patients assessed as ineligible to participate in the trial by the principal investigator for any other reason.

### 2.4 Study procedure

All consenting participants will be subjected to the measurement of vital signs (blood pressure, pulse rate, body temperature), demographic information survey, medical history (past, present, family), physical examination, laboratory tests, and electrocardiogram tests. Eligible participants who meet the inclusion and exclusion criteria will receive a schedule of visits (screening).

At visit 1, the investigator will check the subject's vital signs and test ADAS-cog, K-BDI, BAQ, DMTS (response and accuracy), and fNIRS (Oxy-Hb, deOxy-Hb). The control group will receive acupuncture treatment for 30 minutes, while measuring a simple EEG with NF, and the intervention group will receive acupuncture for a minute while performing the NF training protocol (5 times for 5 minutes, 30–60 seconds of rest between each trial). In the meantime, the investigator will check the subject's BPM and HRV. The investigator will check the subject's adverse reactions and concomitant medications and inform them that cigarettes and caffeine must be avoided 2 hours before the test, as they affect the HRV. From visits 2 to 24, both groups will be treated in the same manner as visit 1, and BPM HRV will be checked in the meantime. The investigator will check the subject's adverse reactions and concomitant medications and inform them in the same manner as on visit 1. At visit 24, the investigator will check the subject's vital signs and test ADAS-cog, K-BDI, BAQ, DMTS (response and accuracy), and fNIRS (Oxy-Hb, deOxy-Hb). Additional visits may be performed at any time if deemed necessary by the investigator's judgment, or following the subject's request. In the event of an unexpected visit, the following tests will be performed: vital signs, checking adverse reactions or concomitant drugs, and laboratory tests. In principle, administration of concomitant drugs will be prohibited during the trial, and when combination therapy may occur, it will be decided whether or not to drop out or exclude at the discretion of the principle investigator (PI). A detailed outline of the schedules is shown in Table 1.

**Table 1:** Schedule of trial visits.

	Study period				
Visit	Screening	1	2 to 24	25	UV
Informed consent	О				
Eligibility screen	О				
Demographic information	О				
Drug history	О				
Medical history	О				
Vital signs	О	О	О	О	О
Laboratory test	О				Δ
EKG	О				Δ
MoCA-K	О			О	
ADAS-cog		О		О	
K-BDI		О		О	
BAQ		О		О	
HRV		О	О	О	
Breath signal of neurofeadback		О	О		
Delayed matching to sample task		О		О	
fNIRS		О		О	
Combined treatment		О	О		
Single treatment		О	О		
Check adverse effects		О	О	О	О
Check concomitant drugs		О	О	О	О
Education of visit schedule	О	О	О	О	О

ADAS-cog = Alzheimer Disease Assessment Scale–Cognitive Subscale, BAQ = body awareness rating questionnaire, EKG = electrocardiogram, fNIRS = functional near-infrared spectroscopy, HRV = heart rate variability, K-BID = Korean version of Beck Depression Inventory, MoCA-K = Korean version of Montreal Cognitive Assessment, UV = unexpected visit.

### 2.5 Intervention

Participants in the control group will visit twice a week for 12 weeks, for a total of 24 visits, to receive acupuncture treatment. Participants in the intervention group will also visit twice a week for 12 weeks, for a total of 24 visits, and receive combined NF-acupuncture treatment. Acupuncture treatment will be performed while abiding by the Standards for Reporting Interventions in Controlled Trials of Acupuncture.^[25]^

#### 2.5.1 Acupuncture method

GV 20, EX-HN1, LI4, and ST36 (total of 9 points) will be selected as intervention points according to their internal agreement with the acupuncture rationale. The specific operation method will be as follows: the subject sits with their back on the bed to conduct NF training or simple EEG together; the needle is inserted perpendicular to the skin at a depth of 5 to 30 mm; Korean Medical Doctor will leave the intervention room after acupuncture in order to blind NF intervention and return to the room 30 minutes later to remove needles.

#### 2.5.2 NF training method

The intervention group will perform the NF sensorimotor rhythm (SMR) training protocol, whereas the control group will have EEG measured for the 30 minutes of acupuncture treatment.

The ProComp 2 Neurofeedback system (Promp Infiniti 8 channel system, Model NO. BA500, Thought Technology Ltd., Quebec, Canada) will be used in this trial. After cleaning the electrode location with an abrasive conductive gel (Nuprep, Weaver and company, CO) and filling the electrode cups with conductive gel (ten 20 conductive paste, Weaver and Company, CO), an electrode will be placed at the subject's central scalp area according to the international 10/20 system. A reference and ground electrode will be placed on the left and right earlobes, respectively. A 3-electrode location input impedance will be maintained under 5 kΩ.^[26]^

The training session will consist of a 6-minute baseline trial and a 30-minute NF training trial, divided into 6 segments. To determine threshold values, each subject will be measured at baseline SMR wave (12–15 Hz, low beta waves) for 3 minutes with eyes closed and 3 minutes with eyes open. NF training, which is designed to increase threshold SMR values, will be performed 6 times for 5 minutes with 20 to 30 seconds of rest between each trial. BPM and HRV values will also be collected in each trial. The threshold value at each trial is newly set according to the subject's feedback.

### 2.6 Sample size

Considering a 10% dropout rate, we conclude that a total of 44 patients is needed to measure the feasibility of combined NF-acupuncture treatment (effect size 0.8, 0.05 significance level in 2-tailed test, 69% power).^[27]^ If the dropout rate is higher than expected, even after deliberation, the number of participants can be adjusted.

### 2.7 Randomization, blinding

The investigator in charge of randomization, who will not be involved in the trial, will generate the random sequence list using SAS Version 9.4 (SAS Institute. Inc., Cary, NC) program, and each randomized code will be kept in sealed envelopes, to be managed by the PI. For randomized allocation, the stratified block randomization method will be used, for which the stratification factors will be gender and age. This method involves stratification into 4 subgroups, by age and gender: 1 group of 50 to 60 years old individuals and 1 of 61 to 75 years old individuals for each gender. The investigator assigning the randomization code will open the sealed envelope in front of the participants to whom the identification code is assigned. This investigator will record the date of opening, sign the envelope, and store it separately.

In this trial, blinding of the subjects is not possible; assessor blinding will therefore be performed to control for bias. An investigator doctor not involved in randomization will perform acupuncture on subjects before NF training or simple EEG, will be blinded to the type of treatment to be administered, and will subsequently perform an evaluation of the effectiveness and safety.

Blinding breaking should be determined on a case-by-case basis and considered only in serious medical emergencies. If the investigator determines that blinding breaking is necessary, they can contact the PI or sponsor to obtain consent, and subsequently document the breaking procedure.

### 2.8 Statistical analysis

All categorical data will be presented as frequencies or percentiles, and will be analyzed by the Pearson chi-squared test or Fisher exact test. All numeric data will be presented as mean, standard deviation, and will be analyzed by Student independent sample *t* test or Wilcoxon rank-sum test to determine normality. Efficacy analysis will be conducted according to the primary full analysis set principle and secondarily by the per protocol principle. All statistical analyses will be based on a two-tailed test and statistically, the significance is set at *P* < .05. The analysis tool will be SAS Version 9.4 (SAS Institute. Inc., Cary, NC). Missing values will be imputed by the last-observation-carried-forward method.

### 2.9 Efficacy assessment

#### 2.9.1 Primary outcome

MoCA-K will be the primary outcome. The endpoint will be the change in the MoCA-K score from screening to visit 25. We will use the covariance analysis model that includes each group as a fixed factor, MoCA-K score as a dependent variable, and the difference in demographic characteristics as a covariate.

#### 2.9.2 Secondary outcomes

ADAS-cog, K-BDI, BAQ, fNIRS, DMTS, BPM, and HRV will be the secondary outcomes. Endpoints will include the change in ADAS-cog, K-BDI, BAQ, DMTS scores, and fNIRS values from visit 1 to visit 25; HRV values from visit 1 to visit 5, 9, 13, 21, and 25; and BMP values from visit 1 to visit 25. As primary outcome, we will use the covariance analysis model that includes each group as a fixed factor, ADAS-cog, K-BDI, BAQ, DMTS scores and HRV, BPM, and fNIRS values as dependent variables, and differences in demographic characteristics as covariates. A paired *t* test or Wilcoxon signed-rank test will be performed on the same participant on the secondary outcome before and after treatment in each group.

#### 2.9.3 Safety assessment and adverse events report

Evaluating changes in laboratory test results from screening to visit 25, checking of vital signs, and interviews about adverse events at each visit will be performed for safety assessment and adverse event reports.

#### 2.9.4 Safety assessment

Assessment subjects will be primarily assigned to intention-to-treat and secondary per protocol groups. Adverse events will be reported as follows: symptoms, onset, duration, severity (mild, moderate, severe), and intervention causality (definitely related, probably related, possibly related, probably not related, definitely not related, unknown).

#### 2.9.5 Adverse events report

PI will educate the investigator, subjects, or authorized surrogate to inform of any reaction that may occur during or after intervention. If adverse events occur, the involved parties will take appropriate action to minimize adverse reactions. For subjects with adverse reactions, follow-up should be performed until symptoms disappear and the condition is stabilized and a progress report should subsequently be submitted. When a serious adverse event occurs, the investigator will immediately report it to the PI. The PI will thereafter suspend all or part of the clinical trial and report it to the Institutional Review Board (IRB) within 24 hours.

### 2.10 Data management

The identification information of all documents related to the trial, including the case report form, will be recorded according to the identification code, and the information will be kept confidential in a locked place even after the results of the clinical research are published. For monitoring and trial process management, sponsors or monitoring staff can access and duplicate the document. A separate center's document storage will be prepared for document security. For 3 years after the completion of the result report, a staff in charge of security will be allocated in the storage.

### 2.11 Monitoring

An independent staff member on the sponsor side can conduct monitoring by calling and visiting. There are three regular visits, but one or two additional visits can be made depending on the speed of recruitment. The specific visit frequency depends on the monitoring Statement of Purpose. When visiting, the staff will check adherence to the registration schedule and compliance with Korea Good Clinical Practice guidelines. The potential presence of omissions will be checked in records by checking all documents and consulting with the investigator if there is any problem.

### 2.12 Ethics and dissemination

This trial will be conducted under the principles of the Declaration of Helsinki, and guidelines of the International Council of Harmonization-Good Clinical Practice guidelines. This study has been approved by the IRB of Daejeon University Daejeon Medical Center (No. DJNP-20–01) on April 22, 2020. Whenever there are important protocol modifications, the revision date, reason, content will be recorded and kept. Moreover, protocol revision should be approved by the IRB, and relevant parties, including investigators and participants, should be informed. Consent documents that include participants or authorized surrogates’ voluntary signatures and signature dates should be obtained by the investigator with sufficient explanation of this trial, the appropriate time to determine consent or assent. The trial result dataset cannot be published or used without the consent of the sponsor.

## 3 Discussion

NF training is used as a treatment for memory deficit worldwide; however, despite several trials indicating its effectiveness, it remains controversial. Acupuncture has also long been used as a treatment for memory deficits; although significant evidence has been accumulated, controversy remains regarding the level of evidence, and its effectiveness is also controversial. This randomized, assessor-blind, pilot trial protocol aims to evaluate the efficacy and safety of NF-acupuncture combined therapy compared to acupuncture single therapy for MND patients.

MoCa-K was developed for the purpose of screening patients with mild cognitive impairment. Compared to other tests, it is more useful for screening patients with mild cognitive impairment as it includes more prefrontal executive function evaluation items.^[28]^ We consider the outcome of this test as the primary outcome in this trial, because it appears useful in evaluating the effect of combined NF-acupuncture treatment.

Although this study is limited in the number of subjects, it is the first randomized controlled trial conducted with NF-acupuncture combined therapy. It will clinically support the wide use of NF training with acupuncture for the treatment of MND. Moreover, we expect that this trial will lead to further study of NF-acupuncture combined therapy or NF training for MND patients.

## Author contributions

All of the authors have read and approved the final manuscript.

**Conceptualization:** Jun-Hwan Lee, In Chul Jung.

**Methodology:** Jieun Kim, Ju-Yeon Kim, Hae Sook Kim, Yang-Chun Park.

**Supervision:** Jun-Hwan Lee, In Chul Jung.

**Writing – original draft:** Jin Hyung Jeong, Changjin Jung.

**Writing – review & editing:** Jin Hyung Jeong, Ju-Yeon Kim.
